# Selections and Abstracts

**Published:** 1901-02

**Authors:** 


### SELECTIONS AND ABSTRACTS.

### Treatment of Stricture of the Rectum.

By George J. Monroe, M.D., Louisville, Ky.

The principal object to be accomplished in the treatment of stricture of the rectum is to relieve pain and suffering, to reduce the indurated condition if we can, to dilate the stricture so the patient can expel the feces without having to strain to exhaustion. If we can do these things we accomplish nearly all that can be done to any advantage. We must use means to make the feces as soft as possible. I believe that a soft stool is better than liquid, and is passed with a greater degree of comfort. We must try and produce absorption of the hard strictured matter. We must try to heal the ulceration. We will find it necessary in close strictures to give something which will relieve pain. Nearly all strictured patients are run down, prostrated, and worn out physically; therefore they require building up by tonics and nourishment. To a great extent we can keep the feces soft by foods. We should also select those foods which only leave a small amount of residual matter. Milk stands at the front of all foods in this respect. Rich soups and broths come next. Eggs, raw or soft boiled, are good. I am in the habit of allowing my patients a little beef or mutton once a day. This should be rare cooked and thoroughly masticated. Patients should avoid peas, beans, corn, cabbage, apples, and, in fact, all foods which produce much gaseous formation.

In combination with foods we will often be compelled to use laxatives. I rely to a great extent on the laxative mineral waters. I find the Apenta one of the best, but I advise that this be greatly diluted with water.

The cascara sagrada answers a good purpose in rendering the feces soft. Cascara does not produce liquid stools. It answers a good purpose also in emptying the rectum without producing or causing very much straining. We can use castor oil and glycerine. Even sweet oil does very well.

As a drink I recommend my patients to use freely the slippery elm water or mucilage. Lemon juice or some slight bitter water may be used to make it more palatable. We should avoid using strong purgative medicines. They do harm by increasing the straining. Emptying the rectum two or three times a week by a liberal injection of slippery elm musilage affords a great deal of comfort, or we may use warm water with glycerine in it, or we may add the glycerine to the slippery elm water. The injection can be used through a bougie provided we have any difficuly in passing it above the stricture.

In regard to the use of antisyphilitic medicines, where syphilis has been the cause of the stricture, I find a difference of opinion. Some very eminent surgeons claim they are beneficial, while others no less eminent say they are of no use. In my own hands I do not believe I have ever seen any benefit result from their use. I have used them in every case of syphilitic stricture I have ever had, but I believe, so far as reducing or producing absorption of the stricture is concerned, they are absolutely useless. In addition to the mercury and iodide of potassa by the mouth, I have smeared the ulceration and stricture with mercurial ointment liberally every day, but have never seen any benefit from it. I have about come to the conclusion that antisyphilitic medicines in this class of cases are useless.

I have had good results from using my fingers or a bougie once a day. The pain is so severe sometimes that we are obliged to use anodynes. Opium, morphine, codeine, hydrate of chloral, hyoscyamus and the bromides may be used. We will have to watch the use of these remedies closely for fear of a habit being produced. I have seen several instances where these narcotics have been used where a habit has been established. I have a case of rectal stricture under treatment now where the gentleman uses a drachm of morphine every five days. In a year from this time, if he lives that long, he will be using twice that amount.

Sometimes relief may be obtained by large hot poultices or cloths wrung out of hot water placed over the anus and abdomen.

Gentle massage, with faradic electricity over the colon, is of service. It will break up hardened feces, increase the flow of intestinal mucus, as well as expel flatus.

We should induce our patient to use all the natural means of health possible. This embraces exercise in the open air, sleeping in well-ventilated rooms, cleanliness, etc. He should be warmly dressed in the winter season. Cod-liver oil combined with Maltine is a good tonic and food for this class of patients. It seems to have a tendency to soften the feces as well as to build up the system. Sometimes iron and the hypophosphites are required. Strychnine and the bitter tonics are usually necessary. Nearly always we have to aid the digestive organs. We may use pepsin, pancreatin, dilute nitro-muriatic acid, etc.

### DILATATION OF THE STRICTURE.

Some means of dilating the stricture is necessary, or at least it is of service. I believe the very best thing we can use for this purpose is the finger. Introduce the index finger until it comes in contact with the stricture. If you can, without using much force, pass it through the stricture, then by passing the finger up and down we massage the contracted part. Keep this up for ten minutes at each treatment. Repeat every day or every other day. This both dilates and produces absorption. I have seen great good done in this way. I have dilated from the size of a lead pencil to the size of my finger in two or three weeks' time. We may use a soft rubber rectal bougie. It is better to commence with one small enough to pass through the stricture without having to use much force, and increase in size as we can. The bougie passing over the indurated surface seems to produce absorption as well as dilatation. By using a bougie gently in this way there is no danger of producing irritation or inflammation. It takes a long time to dilate a stricture in this way, but by this gentle means we allow our patient to be comfortable during the treatment. We can give a patient a bougie and instruct him how to use it himself. This he can pass daily, or every other day, as we direct, and he should once or twice a week visit his doctor.

I do not favor forcible dilatation if the stricture is above the anal inch. If within that distance we can safely dilate without doing damage. I always use my fingers in producing forcible dilatation. We know much better what we are doing with our fingers than we do with any dilating instrument ever devised. I dilate very much in the same way that I do in treating a fissure of the anus. We have many kinds of bougies. My preference is the one made by Mr. Wales. This has an opening through it which we can make use of in injecting medicines into the rectum and colon. There are other soft bougies that taper from one end to the other, conical shaped. These do very well in dilating a stricture. The only objection to them is that we are inclined to use too much force in passing them, and there is danger of producing an inflammation by the pressure we use. I have used both galvanic and faradic electricity in strictures of the rectum. We have bougies that are covered with hard rubber with the exception of the end. There may be an inch or two of the steel that is not insulated.

I am not certain that I have ever derived any benefit from the use of electricity, at any rate not any more than I have from the finger or a soft bougie. Claims have been made that the electric current produces rapid absorption of the induration. I have failed to see it. It may be that I have not used electricity right. I know very little about using this agent. I have discarded its use in the treatment of rectal stricture.

INCISION.

I have cut through strictures a good many times. Pass the index finger of the left hand through the stricture, take a probe-pointed bistoury in the right hand, pass it flatwise np by the finger in the rectum. When you get it above the stricture turn the edge of the knife towards the stricture and cut. We may incise in two or three places. Now pack the cut surface with iodoform or boric acid gauze, also fill the rectum with it. I take this precaution against hemorrhage. I once cut an annular stricture in three places. I did not tampon very carefully. Hemorrhage took place, and before I could see him he nearly bled to death. The tampon can be removed the second day.

If the stricture is extensive or high up in the bowel we take a good deal of risk in incising it. It is liable to bleed. There is danger of septicemia, as it is difficult to drain well. I knew of a case dying from hemorrhage where a stricture was cut high up in the rectum.

After incising a stricture it will be necessary to pass our finger or a bougie for some time. If we do not the healing produces a worse contracted condition than before the operation was performed.

After removing the first dressing I am in the habit of gently passing my finger every day for a week, then every other day for another week, then every third or fourth day for the next two or three weeks. Even after this we had better give the patient a bougie and have him pass it once a week for several months.

PROCTOTOMY.

This is used more in France and Germany than in this country. It is being used to some extent in the United States, but as yet I do not think it is a popular treatment. It is claimed that better drainage can be obtained from this operation than from incising the bowel elsewhere. I believe this is a fact. The operation is performed as follows: Take a straight probe-pointed bistoury, lay it flat upon the index finger of the left hand, introduce the finger until it reaches or passes through the stricture, then cut backwards towards or even to the sacrum; now withdraw the knife, cutting all the tissue outwards and backwards to the coccyx. If there are any arteries that bleed we had better ligate them. We now fill this incision with iodoform gauze, pressing it in with some force. Now place a pad over the anus and fasten on with a T-bandage. This should be dressed every second day. We will have to pass the finger also to prevent contraction from again taking place.

If the wound does not heal well we can use carbolic acid and olive oil, or nitrate of silver, ten grains to the ounce; ichthyol, or Listerine.

The entire strictured portion of the rectum has been excised, and the mucous membrane brought down and stitched to the lower portion of the cut. The stitches are apt to pull out and an ugly sore remains. I do not think it has been a successful operation except in those cases where the stricture is near the anal opening, and when this low other methods of treatment are better.

### COLOTOMY.

Opening the colon where the stricture has closed the rectum, or where it is impossible to expel the feces, affords great relief. We can empty the colon and upper portion of the rectum. We can treat the ulceration through this opening. By wearing a compress we can, to a great extent, prevent the feces from escaping. I have not room in this paper to describe the operation. By referring to Allingham's "Diseases of the Rectum" the method will be found fully and ably discussed.—*Cincinnati Lancet-Clinic.*

---

### SOME OF THE ACCIDENTS FROM THE USE OF THE OBSTETRIC FORCEPS. *

It is undeniable that, however well a man may be informed theoretically upon a given subject, he speaks best from the standpoint of recorded and systematized personal experience. I have sought, therefore, to present you to-night some of my experiences in the use of the obstetric forceps. Having selected this subject, I determined upon the tabulation of my cases of obstetrics since the beginning of practice. This has proved a more arduous task than I supposed, and has in addition been rendered somewhat discouraging from the discovery that the record slips of several hundred of my cases have been unaccountably lost. I have fairly complete reports of 250 of the first obstetric cases, and 287 of the last, but of the intervening 657 all data are lost except those of 50 cases, tabulated in a report to the State Medical Society, in 1890, and the fact of the confinement and the results as to the mothers in the others, and a few other data. All these have been my personal cases from the beginning, and none in hospital practice. In addition there have been a large number in consultation, which have afforded much experience in

* Read before the San Francisco County Medical Society, November 13, 1900, by Henry Gibbons, Jr., M.D., Professor of Obstetrics and Diseases of Women and Children Cooper Medical College.

forceps' use. I may add that of my first 500 cases, 4 of the mothers died, but that since the last of these deaths I have 703 consecutive confinements without a death, notwithstanding a number of cases with convulsions, placenta previa, post partum hemorrhage, adherent placenta, and among the earlier cases, septic infection and peritonitis. It is unnecessary, however, to pursue this part of the subject further in this connection.

In the first 250 cases were 21 of forceps delivery; in the last 287 cases there were 49 in which the forceps were used. While attending the 250 cases, I had also 16 confinements in consultation in which forceps were used; and during the period of the 287 cases, I had in consultation 21 forceps deliveries, or a total of 107 forceps deliveries. Besides these I have imperfect records of 70 other cases in private practice and 24 in consultation, or 201 forceps deliveries in all; and if my ratio of such cases was preserved during the occurrence of the intervening 657 deliveries, it would run the number up to considerably more than 200 forceps deliveries. But, of course, I can only draw upon 107 cases for my conclusions. The average has been about 13 for every 100 labors. Why should I have had but 21 forceps deliveries in 250 cases in early practice, and 49 in 287 cases in later experiences? This is explained, at least in part, by two circumstances. In the last series there was a large excess of primaparæ, and a great advance in the social condition. In the first series there were 67 primaparæ, with 14 forceps deliveries, and 183 multiparæ, with 7 forceps deliveries. In the last series of cases there were 124 primaparæ, with 38 forceps deliveries, and 163 multiparæ, with 11 forceps deliveries. But again the average social condition was vastly different in the two series of cases. In the first there were 143 belonging to the laboring class, and 107 who had not been accustomed to muscular occupations; while in the last series there were only 50 of the former and 237 of the latter social class. It goes without saying that primaparæ require the forceps much more frequently than multiparæ, and that the well-developed muscular woman, accustomed to work, resists the exhausting strain of labor better than her more delicate sister. I hold, then, that the latter series does not show an undue increase in the use of the forceps. In my last 100 deliveries I have had 43

primaparæ and 18 forceps deliveries, with only one death of the child during delivery, and that in a case of puerperal convulsions. Another child had been dead for some days, as the skin was discolored and the cuticle loosened.

CASES OF DELIVERY IN PRIVATE AND CONSULTATION PRACTICE.

| Private. | Number. | Primaparæ. | Multiparæ. | Laboring Class. | Non-laboring Class | Death of Mothers. | Death of Children. | Forceps Cases. |
|---|---|---|---|---|---|---|---|---|
| 1st Series | 250 | 67 | 183 | 143 | 107 | 1 | 9 | 21 |
| 2d Series | 657 | ? | ? | ? | ? | 3* | ? | 70† |
| Last Series | 287 | 124 | 163 | 50 | 237 | 0 | 7 | 49 |
| | ...... | ...... | ...... | ...... | ...... | 4 | ...... | 140 |
| Consultation. | | | | | | | | |
| With 1st Series | 23 | 16 | 7 | ? | ? | 1 | 10 | 16 |
| With 2d Series | 78 | ...... | ...... | ...... | ...... | ...... | ...... | 24† |
| With Last Series | 51 | ...... | ...... | ...... | ...... | ...... | ...... | 21 |
| | | | | | | | | 341 |

In my first 21 forceps cases I find the instruments were required once each for tumor of the sacrum, prolapse of cord, failure of the head to rotate, three times for delay from large child (9, 9, and 12¾ pounds), great exhaustion in one case where post partum hemorrhage occurred, protracted labor (30 hours to three days) in five cases, and inertia in nine cases. Two of the children were stillborn. Five of the children were in occipito-posterior positions. In the consultation cases the forceps were required for puerperal convulsions in two, for prolonged labor (three days) in two, for delay in the second stage (inertia) in five, for great exhaustion in one, face presentation in one, and by reason of haste and anxiety on the part of friends and physicians in five. The forceps had been applied by the attending physicians in the latter cases, and had slipped several

*These all occurred in the first 250 cases of this series.
†All that my imperfect records show. There were undoubtedly more.

times, causing much laceration and destroying at least three of the children. There were seven deaths of the children, one case having to be terminated by craniotomy. In my 49 recent cases 38 were primaparæ and only 11 multiparæ. The positions were, L. O. A., 32; R. O. A., 9; R. O. P., 4; L. O. P., 4. The causes were contracted pelvis with occipito-posterior positions in three; convulsions in four; large child, averaging nine pounds, in twelve; prolonged labor in two; occipito-posterior positions without rotation in three; great exhaustion of mother and child in one,—the mother's pulse falling to 40, and the child's to 90; a dead child in one; a dying child in one; and exhaustion and inertia in twenty-two. One of of the children was dead a week or more before labor, as the head was flaccid, and the cuticle peeled off; two died during labor before the forceps were applied; one, cyanotic at birth, lived about nine hours; one, a 9⅝-pounds child in L. O. P. position in a primaparæ in labor for three days, was lost, if not dead before, by including the loop of the cord between the forceps and its head; one was lost by dragging it through a deformed pelvis; one, in a case of convulsions, after twenty-six hours' labor, was lost by hasty delivery, the cord being short and around the neck. In the interest of the mother it is not probable that any of these children could have been saved. I find that the short forceps were used in three of these cases simply to help the exhausted mother in her last efforts; that the Hodge were used in five cases, and the Eliot in thirty-five, the heads in twenty-five cases having rotated and being near or resting upon the perineum; while in eighteen cases they still remained in the oblique diameters. In six instances the Tarnier forceps were used, the heads being high. The duration of labor was over twenty hours (which is the average) in twenty-five cases, four being two days and more.

In a report to the State Medical Society at its session in 1890, referring to my belief that children average heavier in this State than on the eastern border or in Europe, I gave the average weight of fifty new-born children, in my recent experience, as 7⅝ pounds. The full-term children of the 287 confinements herein reported averaged 7.65 pounds, and of my last 100 cases, in which were 43 which weighed 8 pounds and over, the average was 7.75 pounds.

This circumstance has an important bearing on the question of forceps' use. One hundred and ten children of primaparæ at term averaged 7.41 pounds, while one hundred and forty-three multiparæ averaged 7.81 pounds, an excess of 0.35 pounds in favor of the latter. And again the children of 26 primaparæ averaged 7.47 pounds, while the second children of these same primaparæ averaged 8.27 pounds, or an increase of over ¾ pound. The children of the 49 forceps cases, leaving out two which were premature, averaged for the males 8.21 pounds, and for the females 7.44 pounds, or exactly 8 pounds for both sexes together, which is a third of a pound higher than the average for all the children of the series.

In the State Society paper already referred to, I presented a table, showing the cause of death of the fetus in 500 cases of labor, 34 being in consultation. I enlarge this table with more recent experiences. It now includes the deaths of the children in 159 instrumental deliveries, of which 106 were in my private practice, and 53 in consultation, with causes so far as ascertained.

FORCEPS CASES—DEATHS OF CHILDREN.

| | My own cases. | Consultation cases. | Total. |
|---|---|---|---|
| Dead and macerated | 1 | ...... | 1 |
| Deformed pelvis, dead before forceps used | 1 | ........ | 1 |
| Dead before forceps applied | 1 | ....... | 1 |
| Feeble child, cyanotic, died shortly | 1 | ........ | 1 |
| Convulsions in mother | 2 | 3 | 5 |
| Deformed pelvis | 1 | 1 | 2 |
| Excessive use of ergot | ....... | 1 | 1 |
| Retention of shoulders | ....... | 1 | 1 |
| Brow presentation | ...... | 1 | 1 |
| Occiput posterior positions | 2 | 1 | 3 |
| Cord compressed by forceps | 1 | 2 | 3 |
| Injured by slipping of forceps before I was called | ........ | 3 | 3 |
| Probably injured by forceps, or cord compressed. | 2 | 9 | 11 |
| Total deaths in forceps use | 12 | 22 | 34 |
| Forceps cases | 106 | 53 | 159 |
| Deaths probably from forceps, wholly or in part. | 6 | 15 | 21 |

It must be remembered, however, that the consultation cases were more serious on the average than my own. In all of them the head was high up and not rotated, while in my last series in more than half the cases the head had rotated into the conjugate.

I have, therefore, had 12 deaths of the child in 106 private forceps cases. Four of these occurred before the forceps were applied, hence in but 8 is it possible to attribute the death to the forceps. But labor could not have been terminated without the instruments; therefore, it is not entirely proper to censure them for the result. In consultation cases the termination was much more unfortunate. The forceps seem in part to have been responsible for 15 deaths in 53 cases. It must be remembered, however, that the consultation cases were more serious on the average than my own. In all of them the head was high up and not rotated, while in my last series, in more than half the cases the head had rotated into the conjugate. In several cases serious injury had been done by attempts at delivery before I was called.

This seems a rather long introductory, and may prove longer than that which is to follow, but it is in great part germane to my subject, and will serve me as an illustration for several points.

With ordinary care serious injury to the mother from forceps use is not likely to occur. I have never known a death to take place, nor anything more than laceration of the soft parts. In these days of aseptic methods, death should not occur, but the injury to the parts may lead to important disabilities. The perineum may be extensively lacerated even through the sphincter, which is more likely to occur in occipito-posterior positions, when rotation anteriorly has failed to take place. Nevertheless, when carefully managed, the forceps may be made efficient to prevent laceration. I have no worse results with them than without them, and often have no laceration at all. When serious rupture is threatened, I find a resort to episiotomy or rather division of the larger lips, a most satisfactory procedure. It gives straight incised wounds, which are easily sutured, instead of jagged and uncertain tears, which often extend into the vagina, and are not easy to reach. It is possible for a fold of the bladder to be bruised against the symphysis pubis, and a slough and fistula to follow, although gynecologists assert that such an accident is much more apt to follow long delay in labor. Fistula is far more rare since the more frequent use of the forceps. I have never seen a case. But there is an injury that is not infrequently done by the instruments. It is laceration of the neck of the uterus by attempting to extract the head before full dilatation of the cervix. This matter of determining the dilatation of the cervix is deceptive. The examination should always be made during a pain; otherwise the cervix is often so flaccid as to leave the impression that it is completely dilated when it is far from being so. If the patient's strength is good and there are not other contraindications, the forceps should never be applied until dilatation is complete, especially if the head is high up. There is another possible injury that may be readily overlooked. It is bruising of the tissues covering the inner surface of the pubic arch by the jamming of the head and forceps against it. This occurs when the head is still in the oblique diameter and pretty well up, so that the handles of the ordinary forceps cannot be depressed sufficiently to ensure traction in the direction of the axis. The remedy is a very simple one: that is to use an axis traction forceps. The books tell us that the injudicious use of the forceps may cause separation of the pubic bones at the symphysis, but I apprehend that this accident cannot occur except in those rare cases of great relaxation which prevent locomotion. I have had a few cases of the latter, but none in which forceps were used. Indeed the con dition would be favorable to delivery without the forceps.

The danger to the child is far more serious than to the mother, as our statistics show. Minor injuries are not infrequent, and are of little importance: such as temporary facial paralysis from pressure of the forceps upon the facial nerve below the ear; abrasions and bruising of the face, neck, and scalp; injury to the eyelids, with following conjunctivitis; rupture of the vessels of the scalp, causing hematomata, etc. If the forceps are not adjusted to the sides of the head in the occipito-mental diameter, but are applied obliquely, and much force is used in extracting, there may be very much distortion, with possible fracture of the bones, rupture of vessels in the brain, or laceration of the brain itself. Injuries not apparent at once may result in inflammation later. The skull is so constituted as to permit overlapping of its parietal and frontal bones and diminution of its bi-parietal diameter by a quarter or even half an inch without serious injury, but the pressure must be evenly applied and mainly laterally, and the process must proceed slowly. This constitutes what is called moulding. It produces the temporary dolicho-cephalous head, long and narrow, by shortening the bi-parietal and trachelo-bregmatic diameters and lengthening the occipito-frontal and occipito-mental diameters, which shortly after birth return to their normal dimensions. If labor be unduly hastened we fail to take advantage of this beneficent provision of nature. It is no doubt more scientific to apply the forceps in the occipito-mental diameter of the child, and this can always be done when the head has rotated to the conjugate diameter of the pelvis and often when it is yet in the oblique diameter; but in some cases the operation is difficult or impossible of accomplishment, and then one blade will apply itself in front of the ear and the other behind the ear on the opposite side. The point of this latter blade will frequently project down upon the neck, especially in an occipito-posterior position. In high operations this is almost necessarily the mode of application of the forceps. Now, if the cord be about the neck of the child, which is very often the case, it is almost sure to be compressed, the circulation to the placenta cut off, and the child destroyed. I am certain that this is much more frequently a cause of death from forceps' use than we have been led to believe. My attention was first called to it from finding a loop of cord between the forceps blade and head in an occipito-posterior case, which had been in labor two days and required relief. I have known it to occur in several cases since. The worst feature is that even knowing the danger it is almost impossible to avoid it, since it is quite impossible to push the hand in in advance of the blade to direct its point. The only precaution that I know of is to listen repeatedly to the fetal heart-beat after applying the instruments, and note if it continues in good force or otherwise. If otherwise, the forceps should be removed. The Tarnier blade is less likely to include the cord.

The conditions of election for the forceps are complete dilatation of the cervix; sufficient time for moulding of the head, that it may more easily pass through the pelvis; completion of the long rotation in occiput or chin posterior positions; and rotation of the head into the conjugate diameter of the pelvis. When these conspire the forceps in competent hands have almost no danger for the child; and the danger should not be much even with the tyro, if he has some experience in the use of tools, and is a man of good judgment. But as we recede from these conditions the risk to the child becomes greater and greater. When the head is still in the oblique diameter and not high up, the risk is not greatly increased, provided the instruments are applied to the sides of the head and in its occipito-mental diameter, with the occiput to the heel of the blades. The difficulties of application have, however, increased, and not even an expert can always get them in exact position. If not exactly applied or if applied according to some teaching in the transverse diameter of the pelvis without reference to the head, they grasp the head more or less obliquely, and if much force be required to effect delivery, may distort the head, cut the scalp, even break the skull, or more probably contuse the brain or rupture a blood-vessel within. Besides, the extremity of a blade is apt to extend down upon the neck, and may include the cord, when it is around the neck, as has already been observed. Again, the blades are more likely to slip, and, suddenly losing their hold, pass rapidly through the vagina, and tear the perineum. But the forceps should never slip in this way. There is no excuse for such an accident. I have never had it occur to me. If the instruments are accurately applied, or even somewhat obliquely, and the index of the hand that clasps the lock be extended to touch the presenting part, the operator at once appreciates when slipping begins, and can avoid it. To be forewarned is to be forearmed. All the risks to the child, just mentioned, are increased the higher the head is in the pelvis, with the added danger resulting from the impossibility, with the ordinary forceps, of making traction in the direction of the axis of that part of the pelvis in which the head rests, and of being required to apply extra force, since much is lost by the necessary dragging of the head against the pubic arch instead of under it. The Tarnier forceps are far better suited to such cases, but they are more difficult of application. When the head is at the brim the dangers are supreme. None but an axis traction forceps should be used in such cases.

Now, I have said that one of the conditions of election is that the cervix be fully dilated. One authority has insisted that the forceps should never be applied otherwise. This is too rigorous a dictum. In profound exhaustion with the pulse permanently 120 or more, in puerperal convulsions, in cases of hemorrhage, concealed or inevitable, when the head has remained for some time fixed in the pelvic cavity, and in some cases of inertia, we must apply them in the interests of the mother. But when these conditions are not present, and the mother's strength is good—her pulse not rising above 90 or 100 in the intervals between the pains—we should wait. It should be borne in mind that the duration of normal labor in primaparæ averages twenty hours, and often extends into the second or even the third day without serious inconvenience or harm. But there is still another reason why we should not deliver with undue haste. Nature is wonderfully conservative, and many a case that it seemed impossible to terminate without aid, has, within an hour or so of such decision, made spontaneous changes that soon ended in delivery. It seems to me that we lose sight of the great importance of the moulding of the head already mentioned. Some hours should be given to this, when the head is in the brim or in the oblique diameters, and especially if the occiput be posterior. When heads are large, as we frequently find them in San Francisco, it is the wisest course to let the moulding process proceed to the fullest extent, short of exhaustion of the patient. In my experience the left occipito-posterior position is considerably more frequent than many authorities admit. I have a number of demonstrated cases among my records. The risk of applying the forceps in these cases, before rotation, is very great. In some I have seen rotation spontaneously occurring after hours of delay. In some I have succeeded in rotating the occiput forward with the forceps, and in others I have been obliged to apply the forceps before rotation, turning the occiput into the cavity, and sometimes with disastrous results to the child. In these cases, also, it is of first importance that the physician should be able to make a correct diagnosis of the position, in order that he may understand the cause of the delay, and be content to wait longer, or, if the forceps must be applied, know how to apply them to the best advantage. In too many of the cases the early application of the forceps complicates and masks them for future examination. When the head remains in an oblique diameter for several hours, notwithstanding strong expulsion pains, it sometimes becomes advisable to assist it over its difficulty by traction with the forceps whereupon it will come down easily and rapidly toward the perineum. Then the forceps may be removed, and the unaided forces will often succeed in effecting the delivery.

Occasionally I have felt assured that the delay in the advance of the head was due to the shoulders, which either had not yet sufficiently rotated or were held back by the cervix, contracting somewhat about the neck of the child. Under such circumstances, when the head is well down and rotated into the conjugate and while considerable traction force is yet required, suddenly resistance gives way with a distinct sensation communicated to the hands, as of the child's shoulders slipping past an obstacle.

Possibly, in view of my admission of eighteen forceps deliveries in one hundred consecutive labors, my assertion that I am inclined to be conservative in the use of these instruments, will not be credited. I am conservative at least in regard to the mother's strength, and my good record in that direction will be my justification. There have been some cases in which I have regretted not having used them earlier. I hear of physicians who resort to them much more frequently than here stated.

Perhaps I can not close these remarks better than by reading the following extract from a paper by Dr. Thomas Addis Emmet, in a recent number of the *American Gynecological and Obstetrical Journal:* "At a meeting of this society (American Gynecological Society) in 1878, many of you will recall the reading of a paper on vesico-vaginal fistula. In it I then pointed out that this injury, when from a slough, was the result of delay in delivery, and never from instrumental delivery, and I also proved that where ergot had been employed, the extent of damage in labor was greatly increased. The older members of the society recall the general sentiment existing in the profession at that time against what was termed 'Meddlesome Midwifery,' and they also know how frequently the use of ergot was resorted to for the purpose of terminating tedious labors in preference to the application of the forceps. Few realize that since the publication of that paper a revolution, as a direct consequence, has taken place throughout the world in the practice of obstetrics. The practitioner who now fails to deliver as soon as possible after the head ceases to recede on the subsidence of a pain, would be judged derelict in his duty, and ergot to hasten delivery has gone out of respectable use. Vesico-vaginal fistula, from having been a common result of child-bearing, is now rarely found, and where of necessity a special ward of forty beds was kept always full formerly in the Woman's Hospital, to accommodate cases from every portion of the world, I now often do not see a case there in an interval of several years. During the past year, as an unusual circumstance, I have had two in my service."—*Occidental Medical Times.*

---

### THE TREATMENT OF GONORRHEA.

BY EDWARD MARTIN, M.D.

My purpose in selecting a subject of this kind for consideration before this august and honorable body was based on the assumption that it is a common disease and treated by nearly every one, and not always with entire success. It also seemed not unprofitable to myself to exchange views in regard to the therapeutics of this affection.

Without taking up the subject of etiology or diagnosis or complications, I propose simply to discuss to-night the treatment which has proved most efficacious in my own hands. It might be well to preface my remarks by stating that I have tried to keep my mind free from prejudice. I have gone back again and again. I have taken groups of cases and observed and compared them in considering the use of the internal remedies. Dr. Christian took from 30 to 80 cases similar in nature and tried the ordinary remedies, such as salol, copaiba, cubebs, and sandal-wood oil. The fact was established as far as could be established from this limited experience that cubebs is quite useless, which is fortunate, because it is very expensive. Salol, sandal-wood, and copaiba are all helpful.

The results with my present treatment are based upon trials and re-trials. Such a personal experience may be misleading, and it is in the hope of being corrected from such errors as I may have fallen into that I bring the subject before the Society.

I have nothing to say in regard to prophylaxis, except that the measures customarily used are futile. The diagnosis I always base on microscopic examination, which takes no more than two minutes and is nearly always conclusive.

I believe the most important part of the active treatment is that which is local. When the case is presented in the first few hours, when the symptoms are not extremely severe, and usually the symptoms are severe only in those unfortunates who adopt an ill-directed irritating therapeutics, the treatment consists of hand injections with argol or protargol and irrigations of potassium permanganate.

The hand injection is ordered somewhat as follows:

| | |
|---|---|
| Argol | 6 grains |
| Distilled water | 3 ounces |

The patient is directed to use it with a syringe provided with a soft rubber nozzle, holding not over two drams, and with a piston which fits properly. A few drops of the solution are dropped into the meatus, which is held open, the object being to wash the follicles about the meatus, which are usually not touched at all, since the nozzle passes by. The injection is then gently driven in and held for three minutes. These injections are repeated every two hours during the day. At night and in the morning an irrigation of potassium permanganate 1--6000 is given, the patient being instructed in auto-irrigation. The best apparatus is an ordinary rubber irrigating bag holding a quart, and the nozzle which seems the most serviceable is a hard-rubber flattened cone of such size that it fits into the meatus, and by its expansion holds the fluid in if necessary.

When the disease is confined to the anterior urethra the bag is elevated not over three feet. When the anterior urethra is filled the nozzle is withdrawn and the fluid allowed to flow out—this flushing being repeated till the entire pint of solution is used. The temperature of the solution should be about that of the body, even a little higher. The following prescription is given to the patient:

℞ Kalii permang ........................................ ʒss

Aquæ destil ........................................ f℥iij

Sig. fʒj t. Oj aq.

He is directed to add a teaspoonful to the pint of water, gradually increasing the strength up to two teaspoonsful.

When the bottle of argol solution is half emptied it is again filled with distilled water. When it is again half empty it is filled again. After this the injections are repeated every three or four hours until the bottle is emptied. For six to ten days after the first week the permanganate irrigations are given only at night. If there is no discharge and the patient is perfectly comfortable the permanganate irrigations are used every other night, and in a week's time discontinued.

The internal treatment is directed toward making the urine bland. The patient is directed to take six tablets of citrate of lithia each day, a glass of water with each. For the purpose of rendering the urine antiseptic salol is given in five-grain doses not more than four times a day. The diet is that which best suits the stomach, and, barring obviously indigestible or highly seasoned articles of food, there is no material change made in the ordinary bill of fare. There is no particular restraint enjoined in regard to moderate exercise. A hot bath is directed to be taken every night. It relieves the local congestion and insures sleep. Often, not always, in twelve or fifteen days the attack is cured. Sometimes it is cured in six or eight days; and, when I say "sometimes" I do not mean as a very rare exception, but perhaps in 25 per cent. of the cases. Perhaps an equal number of the cases will be cured in from fourteen to twenty days.

A considerable percentage are not cured in that time. The complications which are so frequent under so-called more conservative treatment almost never occur. It often happens that at the end of ten to fourteen days when the injections are stopped the discharge reappears and contains gonococci. In regard to the further treatment, a resumption of the permanganate irrigations with silver injections increasing the strength of both may cure. That has not been my usual experience. I resume the irrigations but supplant them after a brief course of argol or protargol by astringent injections of the anterior urethra and for this the most satisfactory remedy is a mixture containing zinc sulphate, lead acetate and carbolic acid in water. When the gonococci are present and numerous I use argol and protargol to both the anterior and posterior urethras as long as they persist.

Under that treatment and every other, I have seen them still present after two years, but this is rare. I have seen these cases from well known specialists at home and abroad and I think, therefore, it is safe to say that a certain per cent. are beyond our present reach.

After six or eight weeks of persistent discharge the time has come for exploration of the urethra, for the searching out of the suppurating follicles and ascertaining whether or not the follicles of the prostatic urethra are involved ; they mostly are. For this purpose I use the urethrameter, a dilating instrument. This is introduced for detecting the tender spots. The prostatic urethra is examined in part by the bulbous bougie. The prostate is also massaged and purulent discharge resulting therefrom, found in the urine passed immediately after, is carefully examined.

The reason that the follicles are not cured of their inflammation is that they are full of pus and the irrigating fluid cannot get into them, hence before the irrigating fluid will reach them they will have to be emptied. The anterior follicles will be emptied by passing in the urethrameter or bulbous bougie, and the posterior follicles can be emptied by prostatic massage, so that after eight weeks of persistent treatment comes the treatment of the anterior and posterior follicles followed by irrigation, which is forced back to the bladder. The stream is first allowed to flow against the lips of the meatus, after which the nozzle is held within the meatus until the entire anterior urethra is dilated, and the resistance at the compressor urethræ muscle is overcome ; thus the irrigating fluid distends the empty follicles. Instillations of argol and protargol 1 to 3 per cent. are directed to the prostatic urethra, and lead, hydrastis, and carbolic acid are given as a hand injection. Under this treatment the discharge usually disappears in two weeks, but two or three or four weeks more may be needed, and a number of cases still require three months of treatment.

There is a certain group that persist still beyond this period even for three years, and in regard to the treatment of them perhaps the urethroscope is the most valuable instrument. This instrument I have used much in nearly every one of its varieties, but in the last year have required it much less than ever before. The value of the instrument is limited to those cases characterized polypoid or warty vegetations or isolated persistently suppurating follicles. These unfortunate cases require active cauterization. There remains a small percentage of cases uncured after a thorough trial of all recognized therapeutic agencies. The cases wander from one physician to another, from one city to another, and finally get well in spite of neglect or treatment.—*Proceedings of the Alusa Co. Med. Society.*

---

### SOME NOTABLE GAINS IN MATERIA MEDICA DURING THE NINETEENTH CENTURY.

At present one of the most popular themes of discussion in journals of every kind is the triumphs of the last century over Nature, and their relative importance as compared with those made during the preceding centuries. The general verdict appears to be that in every department of human effort and thought no other century is in any manner comparable with the nineteenth. Indeed, the consensus of opinion is that nothing short of a millennium of preceding time can come anywhere near showing so immense a gain. Some go so far as to hold that it requires the combined gains of all past historic time to match the successes of this one century.

It is a pleasing fact to us that in this swift and certain race for human weal, materia medica and therapeutics, in spite of the pessimistic claims of doubters, have held their own with other departments of modern science along lines of practical research and empiric discovery. So far as generalization and the enunciation of fundamental natural laws are concerned, very little has been done, although even here there has been enough to enable the far-seeing to spell out success for the workers of the twentieth century.

Among the grand problems which the pioneers of therapeutic philosophy are considering, the one that holds out the greatest hope comes from chemistry. As yet practically nothing has been done with it by the therapeutist. Perhaps more than a century of research will be required to gather sufficient facts to render it more than a dream. We refer to the attempt being made to discover the connection between molecular structure and the physiological effects of organic bodies. We know that the carboxyl group always produces a sour taste and that the acidity of organic bodies increases in the ratio of the weight of the molecule to the number of carboxyl groups that it carries. It has raised the question as to whether or not the physiological and therapeutic qualities of every substance are not functions of structure and weight. Mendeleeff's law is the expression of this principle for organic matter.

Spurred on by what is already known in this direction, a host of chemists are at work on this problem because of the promise of fame and fortune which it holds out to them. They are trying to discover what is the structural cause of variations in color, variations in taste, and variations in other properties, in order to know how to command the production of such substances as possess a given set of valuable properties. Recent discoveries in the laws of solubilities are helping in the solution of this problem by showing how many properties are due to what are known as ions, or free atoms and free compound radicals, charged with opposite qualities of electricity. This discovery has taught us that salt does not owe its physiological properties to molecule of sodium chloride, but to free atoms of chlorine and free atoms of sodium charged respectively with positive and negative electricity. The ions, therefore, have to be looked to in the future to give up the secrets of quality in accordance with the periodic law.

A century ago Dr. Benjamin Smith Barton, Professor of Materia Medica in the University of Pennsylvania, wrote in his "Collection for an Essay Towards a Materia Medica of the United States" (*Bulletin of the Lloyd Library of Botany, Pharmacy and Materia Medica*, No. 1, 1900; Reproduction Series, No. 1, xi): "I am not ignorant that there are some persons who consider the science of medicine as a science of extreme simplicity; who believe, or affect to believe, that in the treatment of diseases, we have arrived at something like the *ultimatum* of perfection. We are already, say these persons, in possession of all the means that are necessary for the alleviation, or for the cure, of our diseases. It is needless then to ransack nature any further."

Suppose the men who held such views one hundred years ago should now be resurrected and enabled to realize fully the extent and quality of our medical supplies, what would they be likely to think of them? Let them have placed before them chloroform, ether, cocaine, and the various external and internal antiseptics now at our command, and have them see how we rationally use them. Would it not be a great revelation to them and a complete rebuke to their old-time conceit? Although we have barely made a start along the road of therapeutic progress, there are still many medical men who act as if inwardly convinced of the truth of the fallacy so neatly challenged by Prof. Barton. So numerous and so startling have been the accessions of the past few years that no one now dares for the very shame openly to avow this doctrine. The conduct of these doubters, however, confirms this interpretation of their methods and silence. If pressed to explain their true position, some of the more knowing ones would no doubt confess their skepticism, and, in justification, refer to the apparent fact that most gains now being made in materia medica are within the limits of a few distinct classes of remedies. We have been discovering hypnotics, analgesics, antiseptics, and antipyretics by wholesale, and every eye is fixed chiefly upon these. The men who believe that we are at the end of useful discovery ask us what we are getting beside these. To superficial thinkers this question is difficult to answer, but after a little consideration it is discovered to be pointless. Are we not also getting cough-relieving remedies, substances that stimulate digestion and secretion of digestive fluids, bodies that check hemorrhage through the nervous system without the production of a clot, local anesthetics, tasteless antiperiodics, substances that act antiseptically after reaching the bladder and that relieve the victims of uric-acid poisoning, serums that act as specific destroyers of diphtheria, and last, but not least, glandular secretions, like those of the thyroid gland, that supply deficiencies of the normal constituents of the blood, the absence of which perverts metabolism. Surely these belie the claims of those who hold that discovery is being confined to but a few classes of remedies, however numerous they may be individually.

It is probable that the discovery of new classes of drugs will go on until they become as embarrassing in their numbers as the special new remedies already are. There will not, however, be any reasonable cause for rebellion against such progress. The more there are the more will be the opportunities for natural selection to get in its good work and give us a decided survival of the fittest. A proper censorship, to act as a directing agent protecting the profession from deception, is all that is needed to put everything appertaining to materia medica on a perfectly sound basis. May this censorship be quickly established!—*Merck's Archives.*

---

### ECONOMY IN THE EMPLOYMENT OF COCAINE.

Dr. Wyat Wingrave (*Journal of Laryngology*, vol. xv., No 12) makes some very pertinent remarks on this subject. His experiments in the Central London Throat and Ear Hospital, as reported below, indicate that much weaker solutions of cocaine than are usually employed may be made to do efficient service.

The prevailing high price, with the occasional personal intolerance of cocaine and its salts, suggested experiments with the view of reducing the quantity employed and increasing its efficiency when used in ear, nose, and throat work.

In the first place, it was found that a two per cent. solution of the alkaloid in equal parts of almond and petroleum oil proved an efficient local anesthetic for the examination of, and slight operations upon, the nose and throat—*e.g.*, passing Eustachian catheter, laryngoscopy, exploration and removal of foreign bodies, etc. Applied either by means of an atomizer or a cotton-wool "swab," although apparently taking a little longer to act, its anesthesia is more prolonged than that produced by a watery solution, probably owing to the fact that oil is less readily removed by the flow of secretion.

With regard to the salt, a watery solution containing cocaine hydrochlorate five per cent., and sodium sulphate two per cent., was eventually selected. The addition of the neutral sodium salt decidedly increases the effectiveness of the cocaine, partly by reason of its penetrating power and partly owing to its solvent action upon the globulins and other proteids which occur in the secretions. The former quality is well illustrated in the influence of Müller's fluid on tissues, the chromic salt penetrating readily under the influence of Glauber's salt.

The action of the combined solution proved to be distinctly more rapid, and the anesthesia was as complete, with a proportion of five per cent. as with cocaine in much stronger solutions by itself. Economy is effected, and the risk of constitutional symptoms is minimized.

When either submucous or subcutaneous injection is required, eucaine hydrochlorate (five per cent.) may be combined with two per cent. sodium sulphate with equal advantage; but when used as a surface application to mucous membranes, eucaine should be followed or combined with a solution of suprarenal extract if local ischemia be desired.—*Med. Age.*

---

### THE MOSQUITO FAMILY.

The word mosquito has no scientific import. Derived from the Spanish or Portuguese, it simply means "little fly"; it used popularly to denote a gnat which bites, and most gnats bite when they have a chance. The word is sometimes extended to include certain midges. The Dipterous family Culicidæ, to which this gnat belongs, contains, according to Major Giles, some two hundred and forty-two species, divided among eight genera. The great majority of species (some one hundred and sixty), however, belong to the genus Culex; Anopheles includes thirty, while the remainder are divided among the other six genera, none of which are large. The collections which have recently been made at the British Museum are said to contain ten species of Anopheles new to science, so that if all Major Giles's species are accepted we have a total of some forty species of the genus which has been hopelessly convicted of being the medium by which the malaria parasite is transmitted from person to person.—*Quarterly Review.*

